# Species‐specific responses to combined water stress and increasing temperatures in two bee‐pollinated congeners (*Echium*, Boraginaceae)

**DOI:** 10.1002/ece3.6389

**Published:** 2020-05-20

**Authors:** Charlotte Descamps, Sophie Marée, Sophie Hugon, Muriel Quinet, Anne‐Laure Jacquemart

**Affiliations:** ^1^ Earth and Life Institute–Agronomy UCLouvain Louvain‐la‐Neuve Belgium

**Keywords:** abiotic stress, bee‐pollinated species, floral traits, flowers, increasing temperatures, nectar, water stress

## Abstract

1. Water stress and increasing temperatures are two main constraints faced by plants in the context of climate change. These constraints affect plant physiology and morphology, including phenology, floral traits, and nectar rewards, thus altering plant–pollinator interactions.
2. We compared the abiotic stress responses of two bee‐pollinated Boraginaceae species, *Echium plantagineum*, an annual, and *Echium vulgare*, a biennial. Plants were grown for 5 weeks during their flowering period under two watering regimes (well‐watered and water‐stressed) and three temperature regimes (21, 24, 27°C).
3. We measured physiological traits linked to photosynthesis (chlorophyll content, stomatal conductance, and water use efficiency), and vegetative (leaf number and growth rate) and floral (e.g., flower number, phenology, floral morphology, and nectar production) traits.
4. The physiological and morphological traits of both species were affected by the water and temperature stresses, although the effects were greater for the annual species. Both stresses negatively affected floral traits, accelerating flower phenology, decreasing flower size, and, for the annual species, decreasing nectar rewards. In both species, the number of flowers was reduced by 22%–45% under water stress, limiting the total amount of floral rewards.
5. Under water stress and increasing temperatures, which mimic the effects of climate change, floral traits and resources of bee‐pollinated species are affected and can lead to disruptions of pollination and reproductive success.

## Graphical abstract

Our paper focuses on bee‐pollinated plant species under water stress and increasing temperatures. We observe the whole plant responses including floral traits and nectar reward which are crucial for plant attractiveness. Plants were not affected in the same way, highlighting the complex response to stress. Particularly, changing floral traits and reward may disrupt plant‐pollinator interactions.

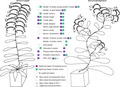

## 1 INTRODUCTION

Drought and above optimal temperatures impair plant growth and development (Lamaoui, Jemo, Datla, & Bekkaoui, 2018), causing more damage in combination than they do individually (Mittler, 2006; Orians, Schweiger, Dukes, Scott, & Müller, 2019; Pandey, Ramegowda, & Senthil‐Kumar, 2015). The frequency of these abiotic stresses increases in the context of climate change (IPCC, 2018). Both stresses affect vegetative and reproductive morphology, physiology, and development (Mittler, 2006; Prieto, Penuelas, Ogaya, & Estiarte, 2008; Rizhsky et al., 2004; Zandalinas, Mittler, Balfagón, Arbona, & Gómez‐Cadenas, 2018). In temperate areas, the majority (78%, Ollerton, Winfree, & Tarrant, 2011) of wild and crop plant species depend on insects for their pollination. Floral attractiveness and visitation rates are presumed to be altered by abiotic stresses linked to climate change, leading to decreased pollination and reproductive success (Borghi, Perez de Souza, Yoshida, & Fernie, 2019; Cohen, Lajeunesse, & Rohr, 2018; Forrest, 2016; Thomson, 2016; Walter, 2018).

The reduced water uptake associated with water stress disrupts plant metabolism. Photosynthesis and physiological processes are affected by water stress, which also reduces leaf number and stomatal conductance, and/or induces stomatal closure (Mittler, 2006). However, metabolic rates increase with increasing temperatures, up to the optimal temperature for a given plant species. Higher temperatures enhance photosynthesis by increasing stomatal conductance (Zandalinas et al., 2018). However, when increasing temperatures and water stress are combined, photosynthetic activity declines for several reasons, including decreased Rubisco activity (Awasthi et al., 2014), damage to photosystem II (Devasirvatham, Tan, & Trethowan, 2016), and increased respiration rate and high leaf temperature (Mittler, 2006). The decreased photosynthetic activity, in turn, reduces the available resources for flower development and reproduction.

Water and temperature stresses are particularly deleterious when they occur early during the reproductive phase and during the blooming period (Barnabás, Jäger, & Fehér, 2008; Scheepens, Deng, & Bossdorf, 2018). The number and size of flowers decrease under water and temperature stresses (Carroll, Pallardy, & Galen, 2001; Descamps, Quinet, & Baijot, 2018; Glenny, Runyon, & Burkle, 2018; Phillips et al., 2018; Takkis, Tscheulin, & Petanidou, 2018). Nectar resources are generally reduced under these conditions, mainly because nectar volume decreases; even when the nectar sugar concentration rises, the total nectar sugar production declines (Carroll et al., 2001; Descamps et al., 2018; Phillips et al., 2018; Takkis et al., 2018). Despite the consequences for food production and wild species survival, studies on floral biology under combined abiotic stresses for bee‐pollinated species are rare.

Our study focused on floral biology modifications to predict the attractiveness of entomophilous plant species under abiotic stresses. We choose two bee‐pollinated, Boraginaceae species: *Echium plantagineum,* an annual, and *Echium vulgare*, a biennial. Both species flowered for at least 5 weeks and produced more than 300 flowers per plant with large amounts of nectar (more than 0.3 mg of sugar per flower), allowing us to easily measure changes in floral biology. To understand the whole‐plant coordinated responses, we compared the physiology, vegetative and reproductive morphology, and nectar reward production of these two species when grown under combined stress conditions (water stress and increasing temperatures). We addressed the following questions: (a) Do the changes in vegetative and reproductive morphology differ between species? (b) Do these modifications lead to a decrease in floral reward production and/or a modification of floral traits and attractiveness for bees?

## 2 MATERIALS AND METHODS

### 2.1 Plant material

*Echium plantagineum* is a late spring annual species native from the South European Mediterranean region. *Echium vulgare* is a biennial or a short‐lived perennial native from the temperate Northern European regions. They are increasingly used in bee‐friendly gardens in temperate Europe. Moreover, *E. plantagineum* is tested in North America as a new crop in support for pollinators in intensive agricultural landscapes (Thom et al., 2016). *Echium plantagineum* develops a 4‐leaf rosette and a branched flowering stem in one season. *Echium vulgare* produces a 20‐leaf rosette during the first year of growth and one flowering stem during the second year (Klemow, Clements, & Threadgill, 2002; Piggin, 1982). Plants of both species are 20–60 cm tall. Axillary stems are produced only in the annual species. The inflorescence and flower morphology are similar. For both species, floral stem develops more than 10 scorpioid cymes which include 20–30 showy 5‐merous campanulate‐tubular flowers. Flowers are hermaphroditic. These two entomophilous species are mainly pollinated by bumblebees, honeybees, and solitary bees (Eberle et al., 2014; Klemow et al., 2002).

### 2.2 Growth conditions

Seeds were provided by Semailles nursery (Faulx‐les‐Tombes, Belgium). Seeds were placed in a germination chamber (Economic Delux model ECD01E; Snijders Scientific) under 20°C/18°C day/night temperature and a 16‐hr light (L):8‐hr dark (D) photoperiod, for 2 weeks. Seedlings were transplanted into pots filled with a 1:1 (v/v) mix of sand (0/5, M Pro) and universal peat compost (DCM). Plants were grown in the greenhouse at the university campus (Louvain‐la‐Neuve 50°39′58′′N; 4°37′9′′E, Belgium) and were watered every 2–3 days with rainwater. Treatments were applied after floral transition under controlled conditions in growth chambers (SEFY platform, Louvain‐la‐Neuve) at different temperature and watering regimes.

To observe the effects of temperature and water stress (and their interaction) on vegetative and reproductive development and photosynthesis‐related parameters, fifteen plants per treatment and species were placed under three temperature regimes (21/19°C, 24/22°C, and 27/25°C day/night) and two watering regimes (well‐watered compared to water‐stressed). The well‐watered plants received daily watering (soil humidity about 25%, as determined using a Procheck Hand‐held Sensor 10 HS moisture sensor, Decagon Devises, Inc), whereas the water‐stressed plants were watered twice a week (soil humidity of 8%–15%). The combination of temperature and watering regimes resulted in six treatments: 21°C well‐watered (21WW), 21°C water‐stressed (21WS), 24°C well‐watered (24WW), 24°C water‐stressed (24WS), 27°C well‐watered (27WW), and 27°C water‐stressed (27WS). In total, 90 plants per species were monitored in three growth chambers. The photoperiod was set to 16L:8D, and relative humidity was maintained at 80 ± 10%. Growth chamber experiments lasted for 6 weeks. Water stress was applied after 1 week of acclimation to the growth chambers; this initial week was considered week 0.

### 2.3 Morphological traits

At week 0, flowering stem height was measured. Every week for 6 weeks, the number of axillary stems (for *E. plantagineum*), new leaves (>2 cm), inflorescences, and flowers at anthesis was counted per plant. At the end of the experiment (week 5), the height of the main flowering stem was measured to calculate the growth rate.

### 2.4 Physiological traits

The 5th‐node leaves of 10 plants per treatment were measured at the beginning of the experiment and 2 weeks after inducing stress. The chlorophyll content index (CCI) was measured using a chlorophyllometer (Opti‐Sciences, CCM‐200), and three measurements were taken per leaf. An automatic porometer (AP4 System, Delta‐T Devices) was used to measure the stomatal conductance. Gas exchange was measured using an infrared gas analyzer (IRGA ADC BioScientific LCI‐SD system, serial No. 33413). The instantaneous water use efficiency (WUE*_i_*) was calculated as WUE*_i_* = *A_i_*/*E_i_*.

### 2.5 Floral and nectar traits

The corolla depth and diameter were measured three times, at weeks 1, 3, and 5, on 10 random flowers in each treatment. In week 3, flowers were dissected, and floral organs were scanned (Ricoh MP C3004 ex PS). The corolla surface area and the length of all stamens per flower were calculated using ImageJ software.

Nectar was extracted with glass capillary tubes (1, 5, or 10 μl, depending on the nectar volume; Hirschmann Laborgeräte) from five flowers per treatment (from five different plants). Total sugar concentration (°Brix) was measured with a low‐volume hand refractometer (Eclipse hand‐held refractometer; Bellingham and Stanley). Nectar sugar content per flower (mg) was calculated following Prys‐Jones & Corbet method (1991).

### 2.6 Statistical analyses

The responses of the two species under both stresses were assessed by Principal Component Analysis (PCA). The normality of the data was estimated using QQ plots and a Shapiro–Wilk test. Physiological and morphological traits were compared between the two species under control conditions (21WW treatment) using a one‐way analysis of variance (ANOVA type I). Results for all treatments were presented as relative differences compared with the control treatment 21WW for each species. The relative difference was obtained by subtracting the value of the 21WW treatment from the value of each treatment, divided by the value of the 21WW treatment. This method allowed a comparison of the responses of both species under the two stresses and their interaction.

To evaluate the effects of water and temperature stresses, linear mixed models and ANOVA type II were performed using three fixed factors (temperature × water × week) and plants as the repeated factor. Linear mixed models were used to analyze repeated measurements over time on the same plants. ANOVA type II was performed to analyze data at each time point. All analyses were performed in R 3.5.2, using the “car” package for *F* test, “lme4” package for linear mixed models, and “FactomineR” package for PCA. Data are presented as means ± standard errors (*SE*).

## 3 RESULTS

### 3.1 Differences in physiology and morphology between the two *Echium* species

**FIGURE 1:**
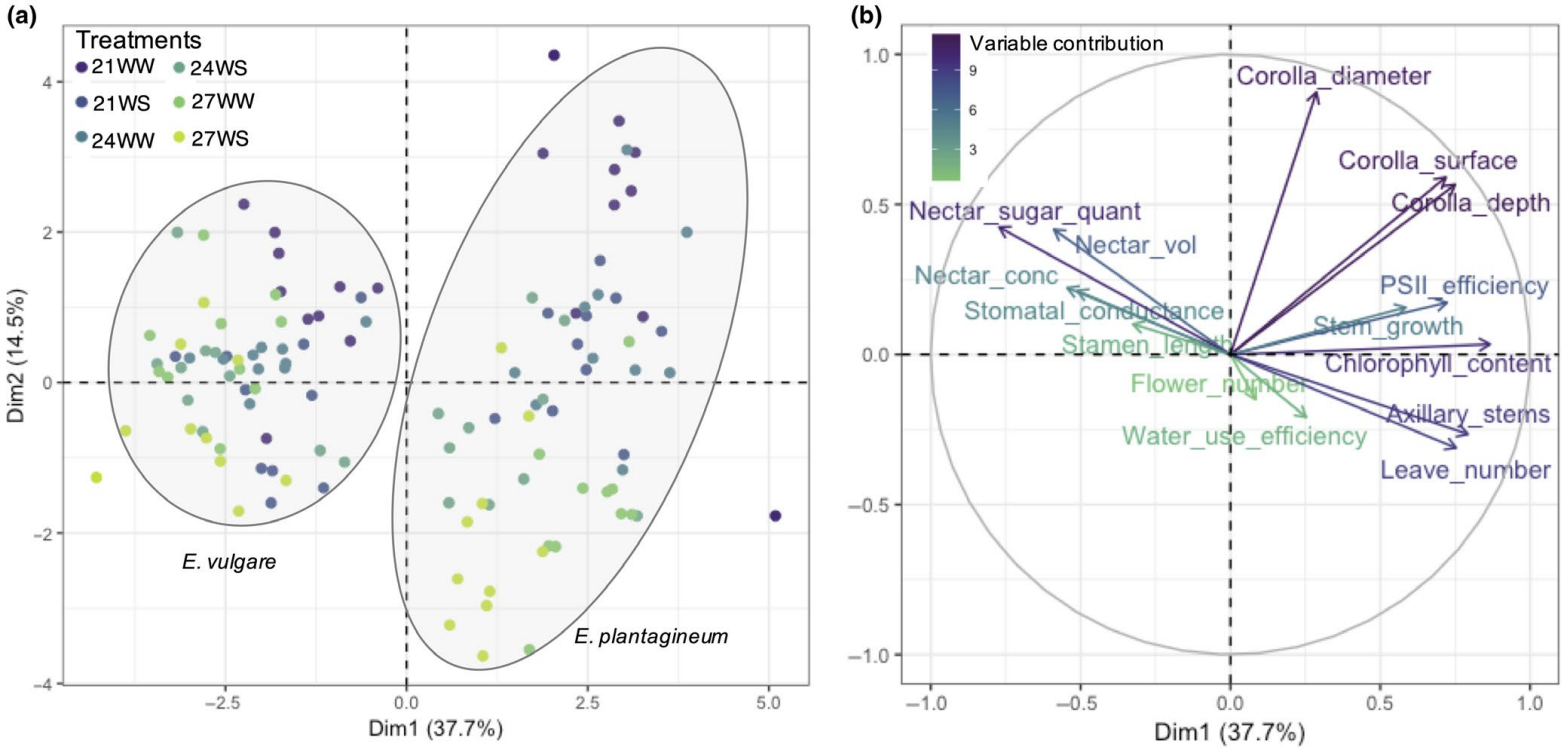
Principal component analysis (PCA) of vegetative, physiological, and floral parameters of *Echium plantagineum* and *Echium vulgare* plants grown under different temperatures (21°C, 24°C, 27°C) and watering regimes (WS, water‐stressed; WW, well‐watered). (a) Individual graph, (b) variable graph of PCA

To obtain a global overview of the responses of the two species to water and temperature stresses, we conducted a PCA of the vegetative, physiological, and floral parameters. The first two axes of the PCA explained 52.2% of the variance (Figure 1). Axis 1 highlighted the differences between the two species and separated them based on differences in physiology (chlorophyll content and PSII efficiency), morphology (leaf number and corolla surface area), and nectar rewards (total sugar content). In the absence of stress (21WW), the annual *E. plantagineum* scored higher than the biennial species *E. vulgare* for morphological characteristics and for some physiological traits (Table 1). The annual species also produced larger flowers than the biennial species, but less nectar with a lower sugar concentration (Table 1).

**TABLE 1:** Descriptive parameters related to morphology, physiology, and floral traits of *Echium plantagineum* and *Echium vulgare* grown at 21°C with watering (*N* = 10 per species)

Descriptive parameter	*Echium plantagineum*	*Echium vulgare*	Species effect^c^
(A) Vegetative traits			
Number of leaves^a^	54.8 ± 7.3	26.0 ± 2.0	*F*_1,18_ = 14.59, ***p = *.001**
Main stem growth rate (%)^b^	154.5 ± 30.0	42.9 ± 13.6	*F*_1,18_ = 11.61, ***p = *.003**
(B) Physiological parameters			
Chlorophyll content^a^	47.1 ± 1.94	22.0 ± 3.94	*F*_1,17_ = 64.16, ***p < *.001**
Photosystem II efficiency^a^	0.87 ± 0.01	0.84 ± 0.01	*F*_1,12_ = 4.98, ***p = *.045**
Stomatal conductance (mmol m^−2^ s^−1^)^a^	153.1 ± 14.7	197.4 ± 35.6	*F*_1,18_ = 2.64, *p = *.12
Water use efficiency (µmol CO_2_ mmol H_2_O^−1^)^a^	1.02 ± 0.16	1.94 ± 0.51	*F*_1,16_ = 5.86, ***p = *.028**
(C) Floral traits			
Flower production^b^	361 ± 65	391 ± 55	*F*_1,18_ = 0.13, *p = *.72
Corolla surface (mm^2^)^a^	515 ± 35	195 ± 8	*F*_1,13_ = 48.05, ***p* < .001**
Stamen length (mm)^a^	19.4 ± 0.9	14.7 ± 0.4	*F*_1,13_ = 14.52, ***p* = .002**
Nectar sugar quantity per flower (mg)^a^	0.4 ± 0.1	1.9 ± 0.2	*F*_1,17_ = 35.92, ***p* < .001**
Nectar volume (µl)^a^	1.8 ± 0.3	3.5 ± 0.6	*F*_1,17_ = 5.85, ***p* = .03**
Nectar concentration (D° brix)^a^	27.6 ± 4.1	47.1 ± 3.6	*F*_1,17_ = 12.13, ***p* = .003**

^a^ 3 weeks of experiment.

^b^ 5 weeks of experiment.

^c^ Significant *p*‐values are in bold font.

Both species showed substantial responses to increasing temperatures and water stress (Figure 1a,b). Axis 2 highlighted the influence of stress on floral parameters when compared to nonstressful conditions. Flower size (diameter, depth, and surface area of the corolla) decreased under both stresses for both species. The *E. plantagineum* response range was broader than that of *E. vulgare*.

### 3.2 The influence of temperature and water stresses on vegetative morphology

**FIGURE 2:**
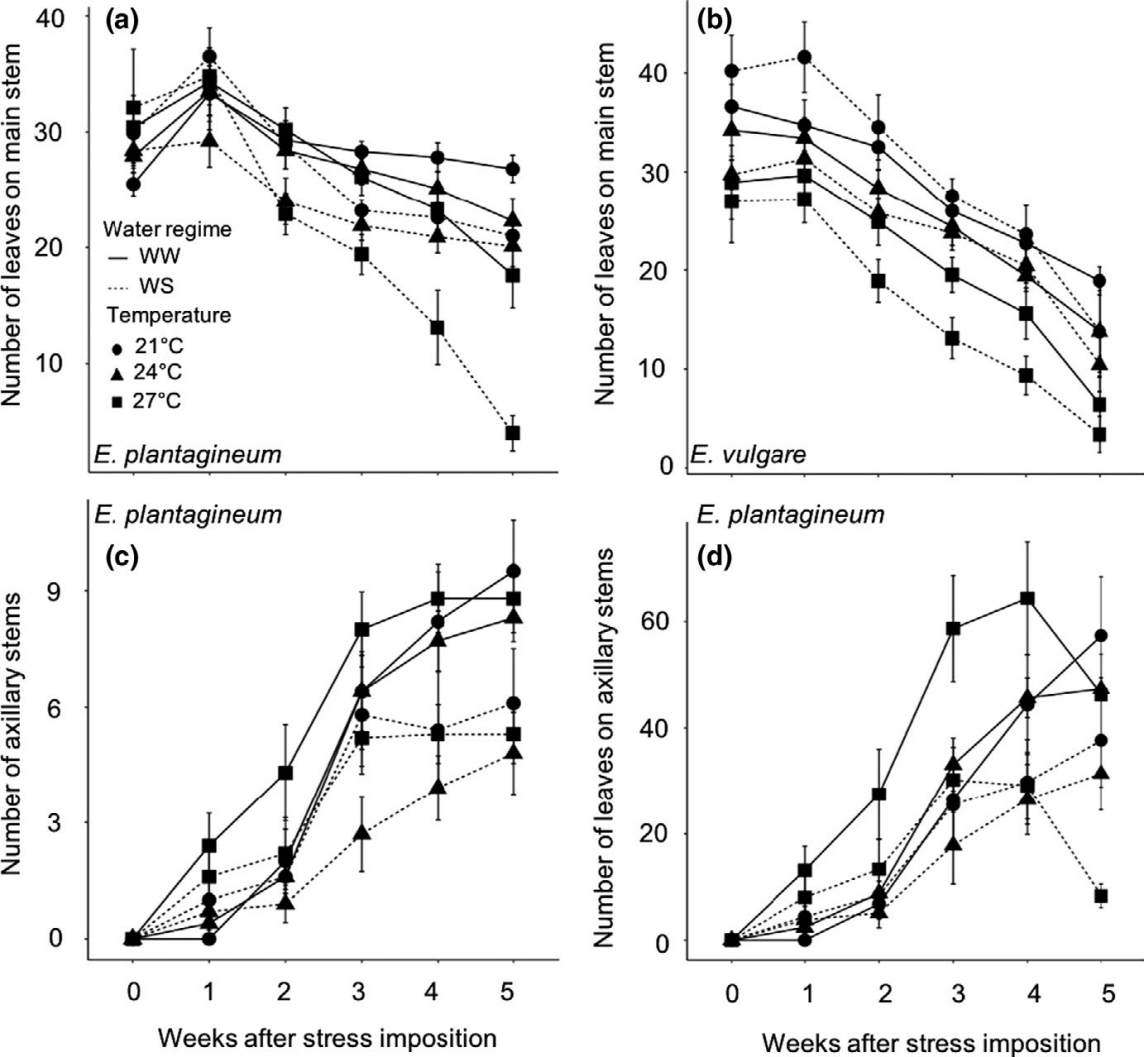
Effects of increasing temperatures and water stress on the number of leaves on the main stem for (a) *Echium plantagineum* and (b) *Echium vulgare*, and on (c) the number of axillary stems and (d) the number of leaves on axillary stems for *Echium plantagineum*. *N* = 10 per treatment in each species. Data are relative values compared with 21WW (means ± *SE*). WS, water‐stressed; WW, well‐watered

We compared the influence of temperature and water stresses on vegetative morphology for both species. The vegetative growth of *E. plantagineum* was negatively affected mainly by water stress (Table 2). The number of leaves on the main stem decreased under water stress and increasing temperatures, particularly at 27WS (Figure 2a), and the growth rate of the main stem was significantly lower for water‐stressed plants (105 ± 19%) than for well‐watered plants (160 ± 32%; Table 2). However, 1 week after the stress imposition, *E. plantagineum* plants still produced axillary stems (Figure 2c) and initiated new leaves on these axillary stems (Figure 2d). Three weeks later, the number of leaves on axillary stems at 27WW was significantly higher than at 21WW (*F*_5,54_ = 3.49; *p* = .008). Thereafter, the number of leaves decreased at 27°C, whereas it continued to increase at 21°C and remained constant at 24°C (Figure 2d). Water stress reduced the number of axillary stems and the number of leaves on those stems at all temperatures (Figure 2c,d).

**TABLE 2:** Statistical results of the effects of increasing temperatures (Temp), water stress (Water), and their interaction (Temp * Water) on vegetative and physiological traits of *Echium plantagineum* and *Echium vulgare*

Parameter	Species	Temp	Water	Temp * Water
Number of leaves on main stem^a^	*Echium plantagineum*	*F*_2,54_ = 6.61, ***p* = .002**	*F*_1,54_ = 17.86, ***p* < .001**	*F*_2,54_ = 2.36, *p* = .10
	*Echium vulgare*	*F*_2,54_ = 25.00, ***p* < .001**	*F*_1,54_ = 1.58, *p = *.21	*F*_2,54_ = 2.06, *p* = .14
Number of axillary stems^a^	*Echium plantagineum*	*F*_2,54_ = 1.67, *p* = .19	*F*_1,54_ = 9.63, ***p* = .003**	*F*_2,54_ = 0.37, *p* = .69
Number of leaves on axillary stems^a^	*Echium plantagineum*	*F*_2,54_ = 1.07, *p* = .35	*F*_1,54_ = 9.53, ***p* = .003**	*F*_2,54_ = 1.58, *p* = .21
Main stem growth rate (%)^b^	*Echium plantagineum*	*F*_2,54_ = 0.12, *p* = .88	*F*_1,54_ = 6.21, ***p = *.02**	*F*_2,54_ = 0.18, *p* = .84
	*Echium vulgare*	*F*_2,53_ = 0.18, *p* = .84	*F*_1,53_ = 0.14, *p = *.71	*F*_2,54_ = 0.46, *p* = .64
Chlorophyll content^c^	*Echium plantagineum*	*F*_2,54_ = 4.88, ***p* = .01**	*F*_1,54_ = 0.40, *p* = .53	*F*_2,54_ = 0.51, *p* = .51
	*Echium vulgare*	*F*_2,49_ = 2.87, *p* = .06	*F*_1,49_ = 12.66, ***p* < .001**	*F*_2,49_ = 0.49, *p* = .62
Photosystem II efficiency^c^	*Echium plantagineum*	*F*_2,24_ = 6.30, ***p* = .006**	*F*_1,24_ = 0.09, *p* = .76	*F*_2,24_ = 0.07, *p* = .94
	*Echium vulgare*	*F*_2,50_ = 3.46, ***p* = .04**	*F*_1,50_ = 0.80, *p* = .38	*F*_2,50_ = 1.41, *p* = .25
Stomatal conductance (mmol m^−2^ s^−1^)^c^	*Echium plantagineum*	*F*_2,54_ = 4.58, ***p* = .01**	*F*_1,54_ = 67.70, ***p* < .001**	*F*_2,54_ = 1.79, *p* = .18
	*Echium vulgare*	*F*_2,51_ = 10.20, ***p* < .001**	*F*_1,51_ = 0.47, *p = *.50	*F*_2,51_ = 1.50, *p* = .23
WUE (A/E) (µmol CO_2_ mmol H_2_O‐−^1^)^c^	*Echium plantagineum*	*F*_2,54_ = 47.23, ***p* < .001**	*F*_1,54_ = 5.54, ***p = *.02**	*F*_2,54_ = 4.90, ***p* = .01**
	*Echium vulgare*	*F*_2,31_ = 3.80, ***p* = .03**	*F*_1,31_ = 0.02, *p = *.88	*F*_2,31_ = 1.56, *p* = .23

Note

Significative *p*‐value is in bold font.

^a^ Linear mixed model (5 weeks of experiment).

^b^ Two‐way ANOVA (week 5).

^c^ Two‐way ANOVA (week 2).

The response of *E. vulgare* plants was different: The number of leaves on the main stem decreased significantly at increasing temperatures but was not affected by water stress (Figure 2b; Table 2). Neither of the stresses influenced stem growth rate, which reached 41 ± 15% regardless of the treatment (5 weeks after stress imposition; Table 2). *Echium vulgare* maintained its growth while exhibiting foliar senescence, whereas *E. plantagineum* exhibited reduced growth and foliar senescence but simultaneously initiated new leaves.

**FIGURE 3:**
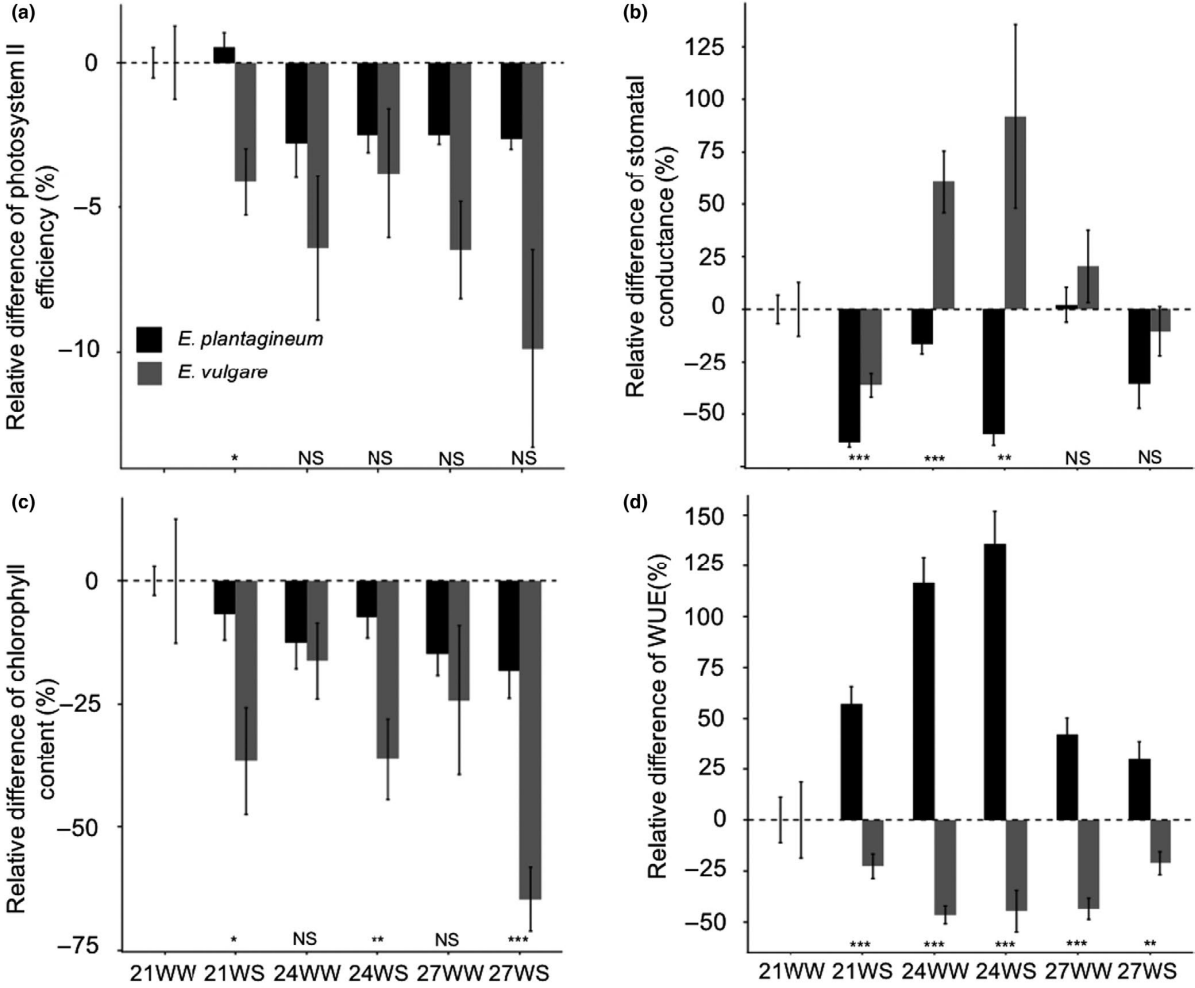
Effects of increasing temperatures and water stress on physiological parameters of *Echium plantagineum* and *Echium vulgare* plants. (a) Photosystem II efficiency, (b) chlorophyll content, (c) stomatal conductance, and (d) water use efficiency 2 weeks after initiating stress treatments. *N* = 10 per treatment in each species. Data are relative values compared with 21WW (means ± *SE*). Species are significantly different (one‐way ANOVA) under the same treatment at *p* < .001 (***), *p* < .01 (**), or *p* < .05 (*). NS, nonsignificant difference. 21 = 21°C; 24 = 24°C; 27 = 27°C; WS, water‐stressed; WW, well‐watered

The two species had different physiological responses to increasing temperatures and water stress. Chlorophyll content was significantly reduced in *E. plantagineum* in response to increasing temperatures, whereas it was significantly reduced in *E. vulgare* in response to water stress (Table 2; Figure 3c). For both species, increasing temperatures but not water stress significantly decreased the efficiency of photosystem II (Table 2; Figure 3a).

Increasing temperatures affected stomatal conductance and water use efficiency (WUE) for both species, whereas water stress significantly affected these parameters only for *E. plantagineum* (Table 2; Figure 3b,d). However, the effects of the stresses differed between the species. WUE increased under stress in *E. plantagineum* and decreased in *E. vulgare* compared to 21WW (Figure 3d). In the two species, both light‐dependent and light‐independent photosynthesis reactions were affected by stresses, and mainly by increasing temperatures.

### 3.3 The influence of temperature and water stresses on reproductive morphology

#### 3.3.1 Floral display and flowering phenology

**FIGURE 4:**
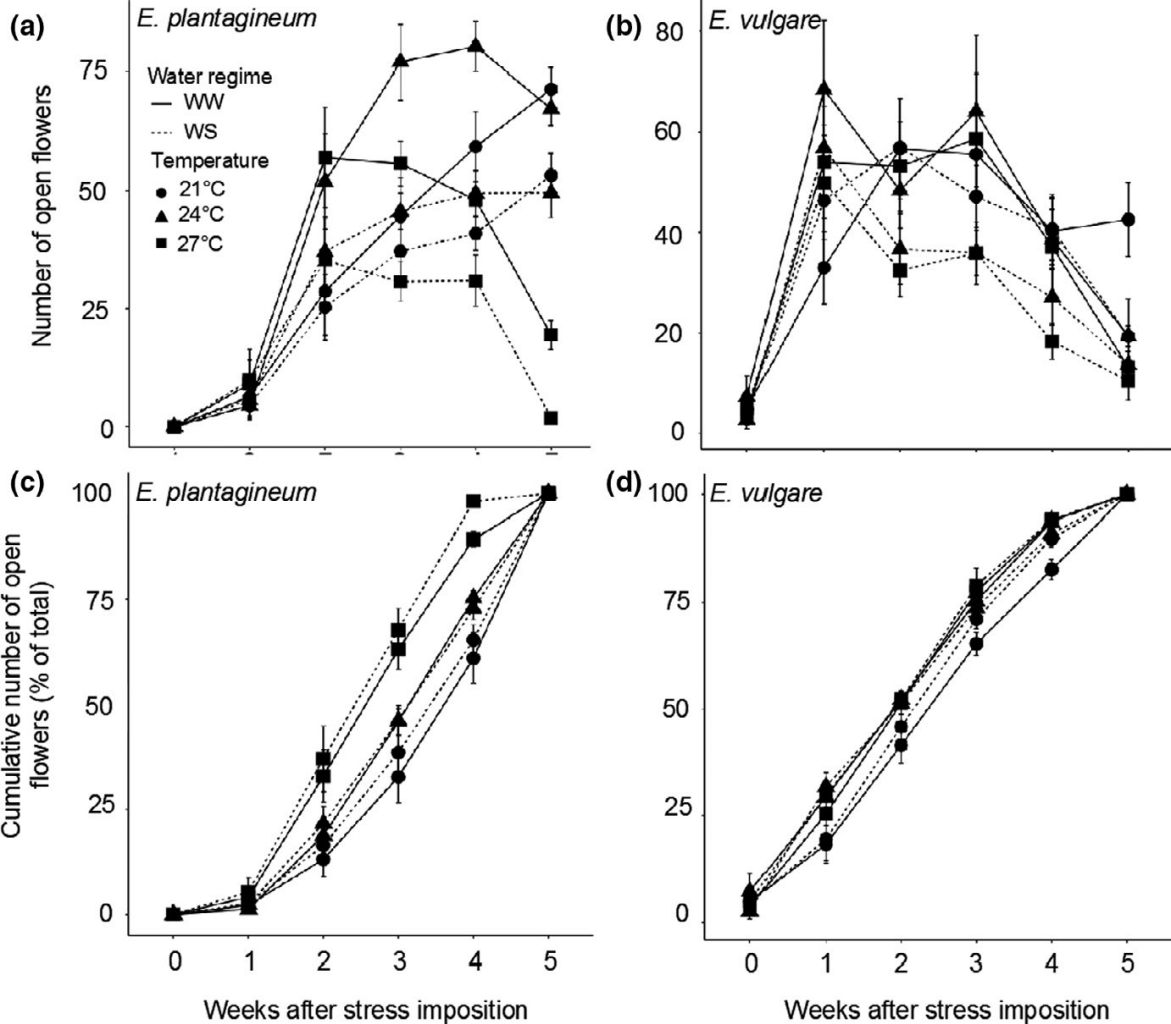
Effects of increasing temperatures and water stress on (a, b) the number of open flowers produced per plant per week and (c, d) the cumulative number of open flowers (% of total) produced per week by (a, c) *Echium plantagineum* and (b, d) *Echium vulgare* plants. *N* = 10 per treatment in each species. Data are relative values compared with 21WW (means ± *SE*). WS, water‐stressed; WW, well‐watered

Water stress reduced the total number of flowers produced in both species and the number of open flowers per plant in *E. plantagineum* (Figure 4a,b; Tables 3 and 4). *Echium plantagineum* produced more flowers at 24°C and 27°C than at 21°C, whereas no significant increase was observed in flower production in *E. vulgare* (Table 3; Figure 4c,d). During the first 2 weeks, stress had little effect on flower production in *E. vulgare* plants, which decreased slightly for 24WS and 27WS treatments from week 3 onwards (Figure 4b). After 5 weeks, all plants were at the end of their flowering period (except for those under 21WW conditions). Flower production in *E. plantagineum* was quite similar in the beginning, regardless of the treatment, but increased sharply at 27°C (Figure 4c). The peak of flowering occurred after 2 weeks for 27WW, after 4 weeks for 24WW, and seemed not to be reached for 21WW, even after 5 weeks (Figure 4a). Although *E. plantagineum* continued flowering after 5 weeks at 21°C and 24°C, it was reduced at 27°C.

**TABLE 3:** Statistical results of the effects of increasing temperatures (Temp), water stress (Water), and their interaction (Temp * Water) on floral traits of *Echium plantagineum* and *Echium vulgare*

Parameter	Species	Temp	Water	Temp * Water
Number of flowers produced after 5 weeks^a^	*Echium plantagineum*	*F*_2,54_ = 15.12, ***p* < .001**	*F*_1,54_ = 27.62, ***p* < .001**	*F*_2,54_ = 1.65, *p* = .20
	*Echium vulgare*	*F*_2,54_ = 0.73, *p* = .48	*F*_1,54_ = 6.31, ***p* = .02**	*F*_2,54_ = 0.41, *p* = .67
Number of open flowers per week	*Echium plantagineum*	*F*_2,54_ = 11.94, ***p* < .001**	*F*_1,54_ = 27.12, ***p* < .001**	*F*_2,54_ = 0.85, *p* = .43
	*Echium vulgare*	*F*_2,54_ = 0.63, *p* = .54	*F*_1,54_ = 3.01, *p* = .09	*F*_2,54_ = 0.38, *p* = .69
Corolla surface (mm^2^)^b^	*Echium plantagineum*	*F*_2,51_ = 42.24, ***p* < .001**	*F*_1,51_ = 17.21, ***p* < .001**	*F*_2,51_ = 0.88, *p* = .42
	*Echium vulgare*	*F*_2,30_ = 1.03, *p* = .37	*F*_1,30_ * = *50.88*,* *** p* < .001**	*F*_2,30_ = 3.05, *p* = .06
Stamen length (mm)^b^	*Echium plantagineum*	*F*_2,51_ = 14.00, ***p* < .001**	*F*_1,51_ = 8.19, ***p = *.006**	*F*_2,51_ = 0.88, *p* = .42
	*Echium vulgare*	*F*_2,30_ = 5.84, ***p* = .007**	*F*_1,30_ * = *41.95, ***p* < .001**	*F*_2,30_ = 0.35, *p* = .71
Corolla depth (mm)^c^	*Echium plantagineum*	*F*_2,52_ = 107.81, ***p* < .001**	*F*_1,52_ = 41.56, ***p* < .001**	*F*_2,50_ = 0.20, *p* = .82
	*Echium vulgare*	*F*_2,53_ = 28.12, ***p* < .001**	*F*_1,53_ = 38.65, ***p* < .001**	*F*_2,53_ = 2.11, *p* = .13
Corolla diameter (mm)^c^	*Echium plantagineum*	*F*_2,50_ = 106.37, ***p* < .001**	*F*_1,50_ = 36.28, ***p* < .001**	*F*_2,50_ = 0.04, *p* = .96
	*Echium vulgare*	*F*_2,53_ = 33.92, ***p* < .001**	*F*_1,53_ = 33.41, ***p* < .001**	*F*_2,53_ = 2.56, *p* = .09
Nectar sugar quantity (mg)^b^	*Echium plantagineum*	*F*_2,51_ = 8.47, ***p* < .001**	*F*_1,51_ = 22.62, ***p* < .001**	*F*_2,51_ = 2.95, *p* = .06
	*Echium vulgare*	*F*_2,52_ = 0.48, *p = *.62	*F*_1,52_ = 3.03, *p = *.09	*F*_2,52_ = 1.54, *p* = .22
Nectar volume (µl)^b^	*Echium plantagineum*	*F*_2,52_ = 5.22, ***p = *.009**	*F*_1,52_ = 34.12, ***p* < .001**	*F*_2,51_ = 2.94, *p* = .06
	*Echium vulgare*	*F*_2,52_ = 0.63, *p* = .54	*F*_1,52_ = 0.11, *p = *.74	*F*_2,52_ = 0.43, *p* = .65
Nectar concentration (°Brix)^b^	*Echium plantagineum*	*F*_2,51_ = 7.88, ***p = *.01**	*F*_1,51_ = 11.36, ***p = *.01**	*F*_2,51_ = 1.26, *p* = .29
	*Echium vulgare*	*F*_2,52_ = 0.13, *p = *.88	*F*_1,52_ = 0.85, *p = *.36	*F*_2,52_ = 0.69, *p* = .51

Note

Significative *p*‐value is in bold font.

^a^ Two‐way ANOVA (week 5).

^b^ Two‐way ANOVA (week 3).

^c^ Linear mixed model (5 weeks of experiment).

**TABLE 4:** Effects of increasing temperatures and water stress on floral traits of *Echium plantagineum* and *Echium vulgare* (3 weeks after stress induction, except for number of flowers produced)

Species	Treat‐ment^a^	Number of flowers produced after 5 weeks^b^	Corolla surface (cm^2^)^b^	Stamen length (mm)^b^	Nectar sugar concentration (D° Brix)^b^	Nectar volume (µl)^b^	Nectar sugar quantity (mg)^b^
*Echium plantagineum*	21WW	361 ± 65 bc	5.15 ± 0.34 a	19.4 ± 0.9 a	27.6 ± 4.1 b	1.80 ± 0.34 a	0.56 ± 0.12 a
	21WS	241 ± 22 c	3.99 ± 0.33 ab	17.6 ± 0.8 ab	35.6 ± 3.1 ab	0.47 ± 0.08 c	0.18 ± 0.03 b
	24WW	511 ± 25 ab	4.26 ± 0.30 a	18.0 ± 0.9 ab	30.1 ± 6.3 b	1.45 ± 0.32 ab	0.36 ± 0.06 a
	24WS	369 ± 30 bc	2.95 ± 0.33 bc	15.3 ± 1.0 bc	47.6 ± 2.7 a	0.21 ± 0.03 c	0.12 ± 0.02 b
	27WW	646 ± 48 a	2.05 ± 0.24 cd	14.5 ± 0.7 bc	20.3 ± 1.2 b	0.61 ± 0.17 bc	0.15 ± 0.05 bc
	27WS	361 ± 65 bc	1.50 ± 0.15 d	12.8 ± 0.8 c	26.5 ± 2.9 b	0.21 ± 0.04 c	0.06 ± 0.01 c
*Echium vulgare*	21WW	391 ± 55 a	1.95 ± 0.08 a	14.7 ± 0.4 a	47.1 ± 3.6 a	3.52 ± 0.59 a	1.83 ± 0.17 a
	21WS	306 ± 37 a	1.07 ± 0.09 bc	10.8 ± 0.5 bcd	43.6 ± 5.1 a	2.87 ± 0.42 a	1.35 ± 0.19 a
	24WW	496 ± 100 a	1.77 ± 0.10 a	13.1 ± 0.8 ab	46.1 ± 3.8 a	2.61 ± 0.30 a	1.36 ± 0.13 a
	24WS	318 ± 38 a	0.98 ± 0.07 c	10.0 ± 0.2 cd	47.8 ± 3.1 a	2.74 ± 0.55 a	1.47 ± 0.20 a
	27WW	559 ± 131 a	1.53 ± 0.06 ab	12.1 ± 0.3 abc	50.9 ± 2.8 a	2.73 ± 0.38 a	1.66 ± 0.22 a
	27WS	330 ± 74 a	1.19 ± 0.12 bc	9.2 ± 0.5 d	42.9 ± 5.4 a	2.88 ± 0.62 a	1.22 ± 0.18 a

Abbreviations: WS, water‐stressed; WW, well‐watered.

^a^ 21 = 21°C; 24 = 24°C; 27 = 27°C.

^b^ Data are means ± *SE* (*N* = 10). Data followed by different letters for each parameter are significantly different (one‐way ANOVA) at *p* < .05 among treatments.

#### 3.3.2 Floral morphology

**FIGURE 5:**
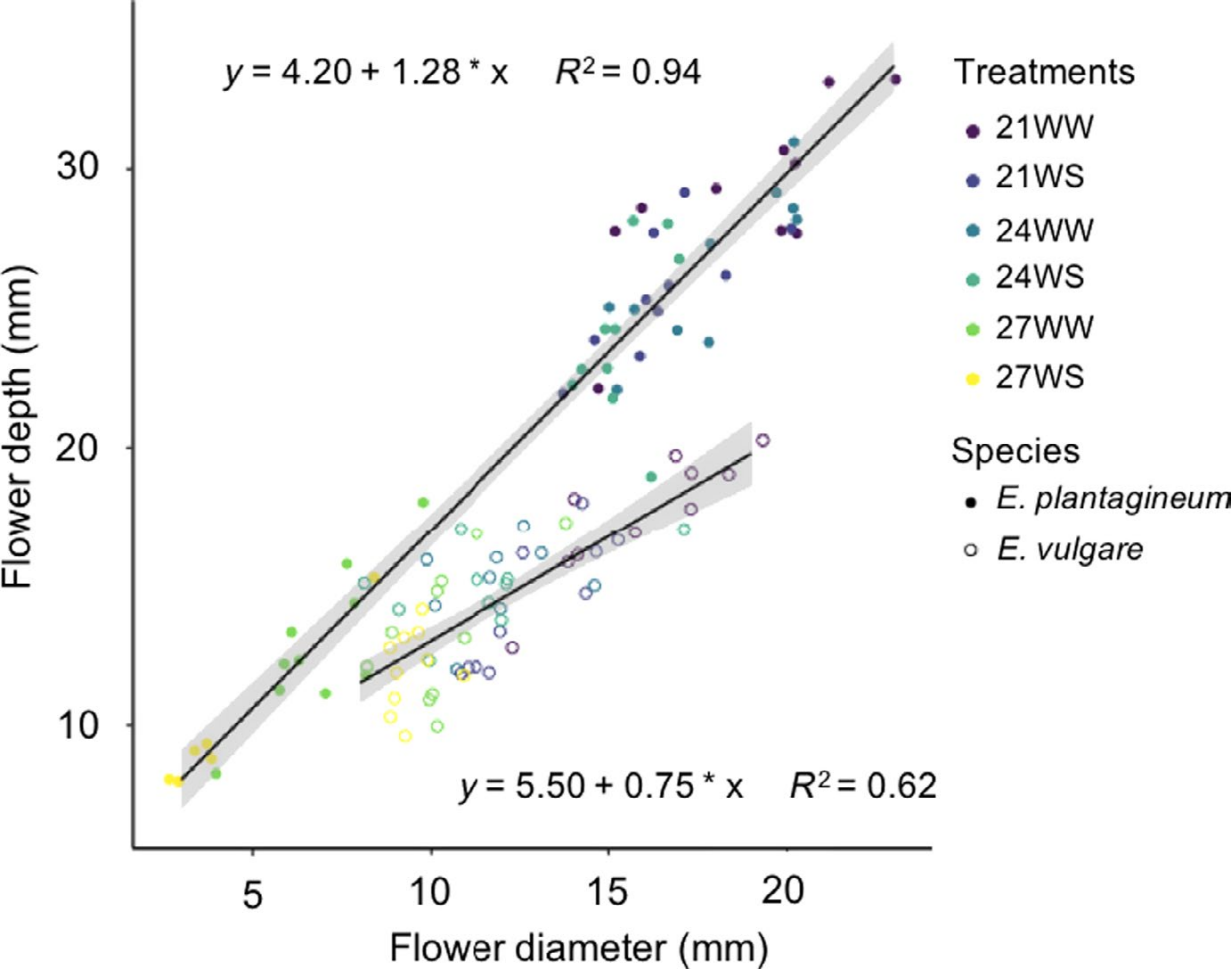
Effects of increasing temperatures and water stress on corolla depth and diameter of *Echium plantagineum* and *Echium vulgare* 5 weeks after stress initiation. *N* = 10 per treatment in each species. The regression equation and *R* ^2^ value are given for each species. 21 = 21°C; 24 = 24°C; 27 = 27°C; WS, water‐stressed; WW, well‐watered

Increasing temperature and water stress had a negative impact on flower morphology in both species. Corolla surface area decreased with increasing temperatures only in *E. plantagineum,* whereas it decreased in both species under water stress (Tables 3 and 4). Under combined water and temperature stress conditions (27WS), the corolla surface area for *E. plantagineum* decreased to about 30% of the control (21WW; 150 ± 15 vs. 515 ± 34 mm^2^) and for *E. vulgare* to about 61% of the control (119 ± 12 vs. 195 ± 8 mm^2^) (Table 4). The mean stamen length was negatively affected by increasing temperatures and water stress in both species: stamen length decreased with increasing stress intensity in *E. plantagineum,* whereas it mainly decreased under water stress in *E. vulgare* (Tables 3 and 4). Corolla depth and diameter were also negatively impacted by both temperature and water stress, with greater reductions in *E. plantagineum* than in *E. vulgare* (Figure 5; Table 3). The range of response was larger in *E. plantagineum* than in *E. vulgare* for floral traits: The difference in corolla surface area, depth, and diameter between the control (21WW) and the most stressful treatment (27WS) was greater for *E. plantagineum* than for *E. vulgare*.

#### 3.3.3 Nectar rewards

Temperature and water stress did not significantly decrease nectar production in *E. vulgare* but did in *E. plantagineum* (Tables 3 and 4). The sugar concentration of *E. plantagineum* nectar increased under water stress and decreased at 27°C compared with the 21WW treatment. However, the nectar volume significantly decreased in *E. plantagineum* flowers under both temperature and water stress. In consequence, nectar sugar content per flower decreased under both stresses in this species (Table 4).

## 4 DISCUSSION

**FIGURE 6:**
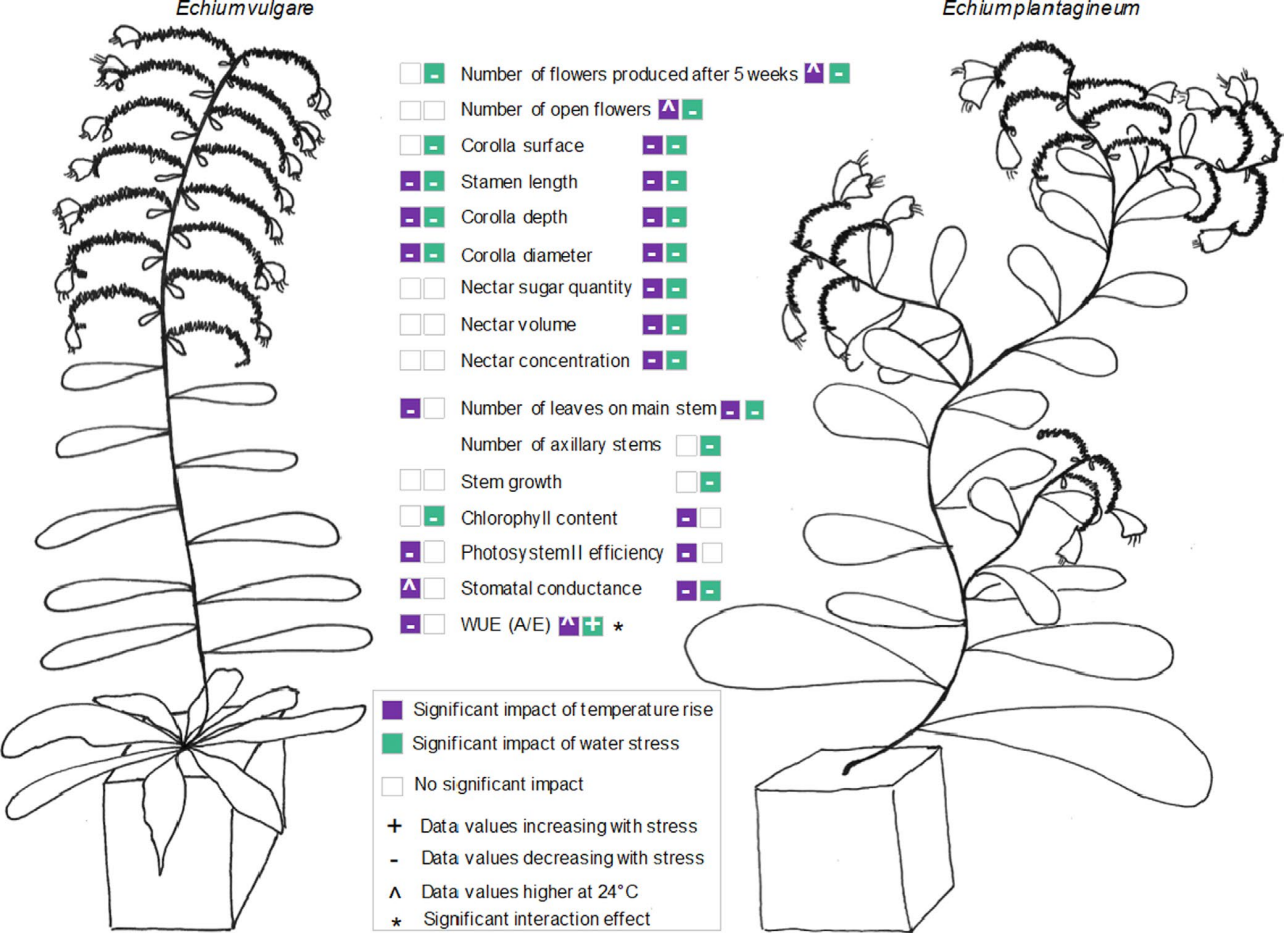
Impact of increasing temperatures and water stress on the morphological, physiological, and floral traits of *Echium plantagineum* and *Echium vulgare* plants grown under different temperatures (21°C, 24°C, 27°C) and watering regimes (WS, water‐stressed; WW, well‐watered). Significant impact means statistically significant impact

The annual species, *E. plantagineum,* was more affected by increasing temperatures and water stress compared to the biennial, *E. vulgare* (Figure 6). For both species, increasing temperatures negatively affected photosynthesis parameters and both stresses reduced flower size. A major difference between the two species concerned nectar production: While it was negatively affected by both stresses for *E. plantagineum*, *E. vulgare* maintained its nectar production under stress.

For both species, increasing temperatures mainly affected photosynthetic reactions. In *E. plantagineum*, both chlorophyll content and PSII efficiency decreased in response to increasing temperatures. However, in *E. vulgare*, only PSII efficiency was affected by increasing temperatures whereas chlorophyll content was reduced by water stress. Even if species were not affected by the same stress, these abiotic stresses compromised the light‐dependent photosynthetic reactions. With respect to light‐independent photosynthetic activity, the two species showed contrasting responses to stress. For *E. plantagineum*, stomatal conductance slightly decreased at 24°C, but drastically decreased under water stress. Reducing stomatal conductance is a mechanism that minimizes water loss and is a common response to water stress in plants that tend to avoid abiotic stresses through physiological adjustments (Lamaoui et al., 2018; Sehgal et al., 2019). Consequently, the WUE was higher for water‐stressed plants compared with well‐watered plants (except at 27°C) and WUE was higher at 24°C compared with other temperatures, suggesting that the plants performed well at 24°C. For *E. vulgare*, stomatal conductance increased at 24°C and no effect of water stress was detected. This species did not close stomata under water stress, suggesting that it has developed osmotic adjustment mechanisms that maintain high water content in the plant without stomatal closure. The two species differ thus in their physiological reaction to these abiotic stresses. Wu, Lowry, Nutter, and Willis (2010) reported that several annual plants had higher WUE in water‐limited environments. Adopting a conservative water use strategy could be advantageous for reproduction in annuals, as their growing period is short.

The decrease in photosynthesis with increasing temperatures was associated with a decrease in the number of leaves on the main stem in both species. At all temperatures, the number of leaves on the main stem decreased over time due to leaf senescence and, for *E. plantagineum*, this effect was reinforced by water stress. Leaf senescence can be induced by temperature and water stresses (Sivakumar & Srividhya, 2016; Wu et al., 2010; Xu & Huang, 2007). However, *E. plantagineum* compensated for this foliar senescence by initiating new leaves on axillary stems, which was not the case for *E. vulgare*. The production of new leaves on the main stem was particularly high in the 27WW treatment between weeks 2 and 4, consistent with previous reports that increasing temperatures can promote leaf development up to a specific optimum temperature (Gray & Brady, 2016).

We observed that increasing temperatures tended to increase the total number of flowers in *E. plantagineum* but did not affect flower production in *E. vulgare*. This result is in contrast to several studies that reported a temperature stress‐induced reduction of flower production for both annual and perennial species (Liu, Mu, Niklas, Li, & Sun, 2012; Takkis et al., 2018). For both *E. plantagineum* and *E. vulgare*, water stress resulted in a decrease in the total number of flowers, and consequently in the overall floral display, with a greater reduction in the annual *E. plantagineum*. Similar results under water stress conditions have been reported in previous studies (Al‐Ghzawi, Zaitoun, & Gosheh, 2009; Phillips et al., 2018). On the contrary, *Mertensia ciliata* maintained its floral display under water stress because this species is able to use stored resources to restart its spring growth; therefore, the effects of water stress are only felt after several consecutive years of drought (Gallagher & Campbell, 2017). Flowering phenology also responded differently to stress in the two species. Phenology was mostly unaffected by stress in *E. vulgare*, compared to the relatively large differences observed between stressed and unstressed plants in *E. plantagineum*. *Echium vulgare* plants, except under 21WW, stopped flowering after 5 weeks, whereas *E. plantagineum* continued flowering after 5 weeks, except in the 27WW and 27WS treatments. *Echium plantagineum* accelerated its life cycle under stress, particularly at 27°C, whereas the *E. vulgare* maintained similar developmental rates under all conditions.

Flower size (corolla surface area, depth, and diameter) was reduced by both stresses in our two species. *Echium vulgare* flowers (at 27WS) were on average two times smaller and *E. plantagineum* flowers five times smaller than control flowers (at 21WW). Reduced flower size (sepals, petals, and stamens) under stress has already been reported for annuals (Descamps et al., 2018; Waser & Price, 2016), biennials, and perennials (Carroll et al., 2001; Gallagher & Campbell, 2017; Halpern, Adler, & Wink, 2010; Opedal, Listemann, & Albertsen, 2016). Producing smaller flowers, which lose less water through transpiration and evaporation, can be advantageous during abiotic stress (Galen, 1999; Halpern et al., 2010).

Nectar volume for water‐stressed *E. plantagineum* plants was on average five times lower (0.30 µl/flower) than that produced by well‐watered plants (1.62 µl/flower at 21°C and 24°C). Several studies have shown that nectar volume decreased in water‐stressed plants (Carroll et al., 2001; Gallagher & Campbell, 2017; Halpern et al., 2010; Waser & Price, 2016). These volume decreases were usually associated with an increase in nectar concentration in water‐stressed plants (Halpern et al., 2010; Takkis et al., 2018). However, several studies reported no rise in sugar concentration under water stress and increasing temperatures (Carroll et al., 2001; Descamps et al., 2018; Gallagher & Campbell, 2017; Mu et al., 2015). At 21°C and 24°C, the WUE of *E. plantagineum* increased and photosynthetic activity was maintained, suggesting that carbohydrate production was also maintained. Even so, the total nectar sugar content produced per plant decreased as stress intensity increased. By contrast, in *E. vulgare*, the nectar rewards (i.e., total sugar content, nectar volume, and nectar concentration) did not change under temperature and water stresses. Phillips et al. (2018) observed similar results for *Lathyrus pratensis*, *Onobrychis viciifolia*, and *Prunella vulgaris*, in calcareous grasslands and attributed the maintenance of the nectar rewards to resistance to water stress for these species in this type of habitat. These results indicate that *E. plantagineum* and *E. vulgare* have different strategies for facing abiotic stress. This difference may be explained by life history traits. Biennials have the option to allocate all their resources to vegetative development during the first year; during the second year, all first‐year resources can then be invested in reproductive development.

Under increasing temperatures, both species produced smaller flowers and fewer flowers per plant. However, floral display and flower size are signals for pollinators. Decreased flower size can reduce flower attractiveness and consequently insect visitation rates and pollination success (Al‐Ghzawi et al., 2009). Moreover, the reduced size of the stressed flowers of *E. plantagineum* was so substantial that it may cause a morphological mismatch with the pollinators. Long tongued (>10 mm long) bumblebee species (e.g., *Bombus pascuorum* and *B. hortorum*) are among the main pollinators of *Echium* spp. (S. Marée, personal com.). Reduced corolla size and tube depth could alter the efficiency of the visitors (Miller‐Struttmann et al., 2015). Furthermore, if floral display is reduced, plant attractiveness is reduced. Nectar production of *E. plantagineum* was reduced by abiotic stresses making the species more vulnerable to pollination disruption than *E. vulgare*. Because nectar rewards attract pollinators, reduced quantities of nectar could decrease attractiveness, visitation rates, and pollination success. Such disruptions in plant‐pollinator interactions include both morphological (corolla size and depth) and recognition (attractiveness linked to nectar production, VOCs emission) mismatches (Gérard, Vanderplanck, Wood, & Michez, 2020).

## CONFLICT OF INTEREST

None declared.

## AUTHOR CONTRIBUTIONS

**Charlotte Descamps:** Conceptualization (equal); Data curation (equal); Formal analysis (equal); Methodology (equal); Software (equal); Visualization (equal); Writing‐original draft (equal); Writing‐review & editing (equal). **Sophie Marée:** Data curation (equal); Formal analysis (equal); Investigation (equal); Methodology (equal); Visualization (equal). **Sophie Hugon:** Data curation (equal); Formal analysis (equal); Investigation (equal); Methodology (equal); Visualization (equal). **Muriel Quinet:** Conceptualization (equal); Investigation (equal); Project administration (equal); Resources (equal); Supervision (equal); Validation (equal); Writing‐original draft (equal); Writing‐review & editing (equal). **Anne‐Laure Jacquemart:** Conceptualization (equal); Funding acquisition (equal); Investigation (equal); Project administration (equal); Resources (equal); Supervision (equal); Validation (equal); Writing‐original draft (equal); Writing‐review & editing (equal).

## Data Availability

Descamps, Charlotte *et al*. (2020), Species‐specific responses to combined water stress and increasing temperatures in two bee‐pollinated congeners (Echium, Boraginaceae), Dryad, Dataset, https://doi.org/10.5061/dryad.9w0vt4bc0
